# Supplementary material for: Incidence of Cardiovascular Disease in Patients with Familial Hypercholesterolemia Phenotype: Analysis of 5 Years Follow-Up of Real-World Data from More than 1.5 Million Patients
Source: J Clin Med. 2019 Jul 23;8(7):1080. doi: 10.3390/jcm8071080 (PMC6678686; doi:10.3390/jcm8071080)
Supplement: Supplementary file 1 [file jcm-08-01080-s001.pdf]

Supplemental Material:

1. **Table S1.** Comparison of Participants Characteristics between population with and without complete pre-treated LDL data in both Familial Hypercholesterolemia Phenotype and Normal LDL-C population groups.
2. **Table S2.** Incidence rate (1000 person-years) and hazard ratio of atherosclerotic cardiovascular disease by age in primary prevention. Complete-case analysis.
3. **Table S3.** Incidence rate (1000 person-years) and hazard ratio of atherosclerotic cardiovascular disease by age in secondary prevention. Complete-case analysis.
4. **Table S4.** Incidence rate (1000 person/years) and hazard ratio of coronary heart disease by age in primary prevention
5. **Table S5.** Incidence rate (1000 person/years) and hazard ratio of coronary heart disease by age in secondary prevention
6. **Figure S1.** Incidence rate (per 1000 person-years) of CHD in primary prevention in FH-P vs normolipidemic population based on the presence of number of cardiovascular risk factors
7. **Figure S2.** Incidence rate (per 1000 person-years) of CHD in secondary prevention in FH-P vs normolipidemic population based on the presence of number of cardiovascular risk factors and clinical associated characteristics
8. ICD-9 and ICD-10 codes included in the definition of ASCVD (coronary heart disease, ischemic stroke and peripheral artery disease)

Table S1. Comparison of Participants Characteristics between population with and without complete pre-treated LDL data in both Familial Hypercholesterolemia Phenotype and Normal LDL-C population groups.

	**Normal LDL-C population (<115 mg/dL)**			**Familial Hypercholesterolemia Phenotype**		
**Variable**	**Complete-case**	**Imputed-case**	**p-value**	**Complete-case**	**Imputed-case**	**p-value**
**N**	490,959	23,218		2,652	10,171	
**Age, mean (SD)**	49.3 (19.9)	71.2 (10.9)	<0.0001	51.7 (15.5)	64.4 (11.5)	<0.0001
**Sex (male), %**	42.2%	56.7%	<0.0001	44.3%	44.9%	0.6126
**Receiving lipid lowering therapy, %**	2.3%	100.0%	-	46.8%	100.0%	-
**Diabetes mellitus, %**	15.1%	50.9%	<0.0001	11.5%	27.7%	<0.0001
**Hypertension, %**	35.9%	89.7%	<0.0001	39.6%	73.3%	<0.0001
**Current smoker, %**	31.1%	37.5%	<0.0001	41.7%	33.6%	<0.0001
**Body mass index, mean (SD)**	27.6 (5.6)	29.5 (4.8)	<0.0001	28.1 (4.8)	29.2 (4.6)	<0.0001
**Obesity, %**	21.8%	40.5%	<0.0001	23.4%	34.5%	<0.0001
**TC, mg/dL, mean (SD)**	169.5 (26.0)	162.7 (34.4)	<0.0001	314.6 (73.3)	236.0 (43.2)	<0.0001
**LDL-C, mg/dL, mean (SD)**	91.8 (16.9)	81.9 (25.3)	<0.0001	236.3 (65.8)	155.8 (37.2)	<0.0001
**HDL-C, mg/dL, mean (SD)**	55.8 (16.4)	51.1 (15.5)	<0.0001	54.8 (14.4)	55.8 (13.7)	0.0049
**TG, mg/dL, mean (SD)**	111.1 (73.4)	143.1 (95.2)	<0.0001	162.3 (106.5)	146.0 (81.2)	<0.0001
**Creatinine, mg/dL, mean (SD)**	0.9 (0.4)	1.0 (0.6)	<0.0001	0.9 (0.3)	0.9 (0.3)	0.0041
**HBA1c, mean (SD)**	5.8 (1.5)	6.4 (1.5)	<0.0001	6.0 (2.0)	6.1 (1.5)	0.5010

SD: standard deviation; BMI: Body Mass Index; TC: Total Cholesterol; LDL-C: Low-density lipoprotein cholesterol; HDL-C: High-density lipoprotein cholesterol; TG: triglycerides; HbA1C: Glycosylated haemoglobin.

Table S2. Incidence rate (1000 person-years) and hazard ratio of atherosclerotic cardiovascular disease by age in primary prevention. Complete-case analysis.

	Normal LDL-C population (<115 mg/dL)			Familial Hypercholesterolemia Phenotype			
**Age Groups**	**Number (n)**	**Events (n)**	**IR (1000 p-y) 95%CI**	**Number (n)**	**Events (n)**	**IR (1000 p-y) 95%CI**	**HR 95%CI**
**All**	459,934	14,574	6.6 (6.5-6.7)	2,489	132	11.2 (9.3-13.4)	2.11 (1.76-2.53)
**0-35**	141,845	142	0.2 (0.2-0.2)	433	5	2.3 (0.9-5.8)	9.20 (3.59-23.53)
**35-45**	91,022	476	1.1 (1.0-1.2)	473	17	7.3 (4.4-12.0)	4.69 (2.81-7.83)
**45-55**	69,489	1,205	3.5 (3.3-3.7)	532	29	11.4 (7.7-16.7)	2.59 (1.75-3.82)
**55-65**	54,442	2,433	9.3 (8.9-9.7)	586	39	14.1 (10.1-19.6)	1.83 (1.31-2.55)
**65-75**	46,720	3,779	17.5 (16.9-18.1)	282	20	15.6 (9.8-24.9)	1.13 (0.71-1.80)
**75-85**	40,631	4,689	27.4 (26.6-28.2)	158	19	29.2 (18.2-46.8)	1.36 (0.85-2.19)
**>85**	15,785	1,850	35.6 (33.9-37.3)	26	3	31.7 (8.4-120.2)	0.98 (0.26-3.69)

LDL-C: Low-density Lipoprotein Cholesterol; IR 1000 p/y: Incident Rate persons-years; CI: Confidence Interval; HR: Hazard Ratio.

Table S3. Incidence rate (1000 person-years) and hazard ratio of atherosclerotic cardiovascular disease by age in secondary prevention. Complete-case analysis.

	Normal LDL-C population (<115 mg/dL)			Familial Hypercholesterolemia Phenotype			
**Age Groups**	**Number (n)**	**Events (n)**	**IR (1000 p-y) 95%CI**	**Number (n)**	**Events (n)**	**IR (1000 p-y) 95%CI**	**HR 95%CI**
**All**	31,025	9,152	85.6 (83.8-87.5)	163	57	100.8 (76.6-132.8)	1.42 (1.07-1.88)
**0-35**	253	20	16.8 (10.6-26.7)	3	2	342.9 (80.1-1466.9)	25.48 (4.47-145.33)
**35-45**	616	111	40.8 (33.6-49.6)	6	3	133.5 (35.8-497.4)	2.85 (0.74-11.01)
**45-55**	1,800	464	62.7 (57.0-69.0)	23	8	90.9 (43.6-189.2)	1.26 (0.60-2.66)
**55-65**	4,228	1,202	71.1 (67.0-75.4)	45	16	100.7 (59.8-169.7)	1.61 (0.94-2.75)
**65-75**	7,169	2,259	83.6 (80.0-87.3)	38	12	80.5 (44.2-146.6)	1.07 (0.59-1.95)
**75-85**	10,961	3,544	98.0 (94.6-101.4)	36	12	109.3 (58.1-205.6)	1.33 (0.71-2.50)
**>85**	5,998	1,552	100.2 (95.1-105.6)	13	5	122.4 (44.9-333.4)	1.45 (0.53-3.96)

LDL-C: Low-density Lipoprotein Cholesterol; IR 1000 p/y: Incident Rate persons-years; CI: Confidence Interval; HR: Hazard Ratio.

Table S4. Incidence rate (1000 person/years) and hazard ratio of coronary heart disease by age in primary prevention

	Normal LDL-C population (<115 mg/dL)			Familial Hypercholesterolemia Phenotype			
**Age Groups**	**Number (n)**	**Events (n)**	**IR (1000 p/y) 95%CI**	**Number (n)**	**Events (n)**	**IR (1000 p/y) IC 95**	**HR IC 95**
**All**	472,591	4,728	2.07	10,621	298	5.8	1.95 (0.65-2.28)
**0-35**	141,920	40	0.02 (0.01-0.03)	551	5	1.7 (0.7-4)	16.70 (6.66-41.85)
**35-45**	91,274	180	0.07(0.06-0.11)	833	19	4.5 (2.9-7.1)	5.74 (3.59-9.20)
**45-55**	70,331	466	0.3 (0.2-0.3)	1,652	28	3.4 (2.3-5.0)	1.81 (1.22-0.68)
**55-65**	56,835	894	0.9 (0.80-1.08)	3,241	89	5.6 (4.59-6.9)	1.85 (1.50-2.28)
**65-75**	50,897	1,284	1.9 (1.7-2.2)	2,713	90	6.8 (5.3-9.0)	1.46 (1.11-1.91)
**75-85**	44,604	1,397	3.2 (2.7-3.8)	1,441	57	8.5 (5.9-12.2)	1.30 (0.90-1.89)
**>85**	16,730	467	3.5 (2.5-4.9)	189	11	1.4 (7.1-29.3)	1.81 (0.89-3.68)

LDL-C: Low-density Lipoprotein Cholesterol; IR 1000 p/y: Incident Rate 1000 persons-years; CI: Confidence Interval; HR: Hazard Ratio

Table S5. Incidence rate (1000 person/years) and hazard ratio of coronary heart disease by age in secondary prevention

	Normal LDL-C population (<115 mg/dL)			Familial Hypercholesterolemia Phenotype			
**Age Groups**	**Number (n)**	**Events (n)**	**IR (1000 p/y) 95%CI**	**Number (n)**	**Events (n)**	**IR (1000 p/y) IC 95**	**HR IC 95**
**All**	41,585	4,579	28.2	2,202	322	34.5	1.29 (1.08-1.53)
**0-35**	262	8	6.3 (3.3-11.9)	8	2	74.5 (18.06-307.6)	5.98 (0.81-44.06)
**35-45**	697	65	19.9 (15.9-25.0)	36	8	54.2 (26.1-112.1)	1.97 (0.93-4.20)
**45-55**	2,329	278	26.5 (23.6-29.7)	214	41	44.2 (32.1-60.8)	1.55 (1.11-2.17)
**55-65**	5,898	687	26.1 (24.2-28.2)	581	80	30.8 (23.4-40.7)	1.22 (0.91-1.63)
**65-75**	10,340	1,236	26.4 (26.49-29.7)	718	101	32.5 (26.2-40.2)	1.25 (1.00-1.56)
**75-85**	14,965	1,765	32.3 (29.8-32.8)	544	76	34.8 (26.8-45.3)	1.19 (0.90-1.57)
**>85**	7,094	540	26.1 (24.09-28.3)	100	13	36.6 (20.1-66.2)	1.49 (0.80-2.76)

LDL-C: Low-density Lipoprotein Cholesterol; IR 1000 p/y: Incident Rate 1000 persons-years; CI: Confidence Interval; HR: Hazard Ratio

Fig S1. Incidence rate (per 1000 person-years) of CHD in primary prevention in FH-P vs normolipidemic population based on the presence of number of cardiovascular risk factors

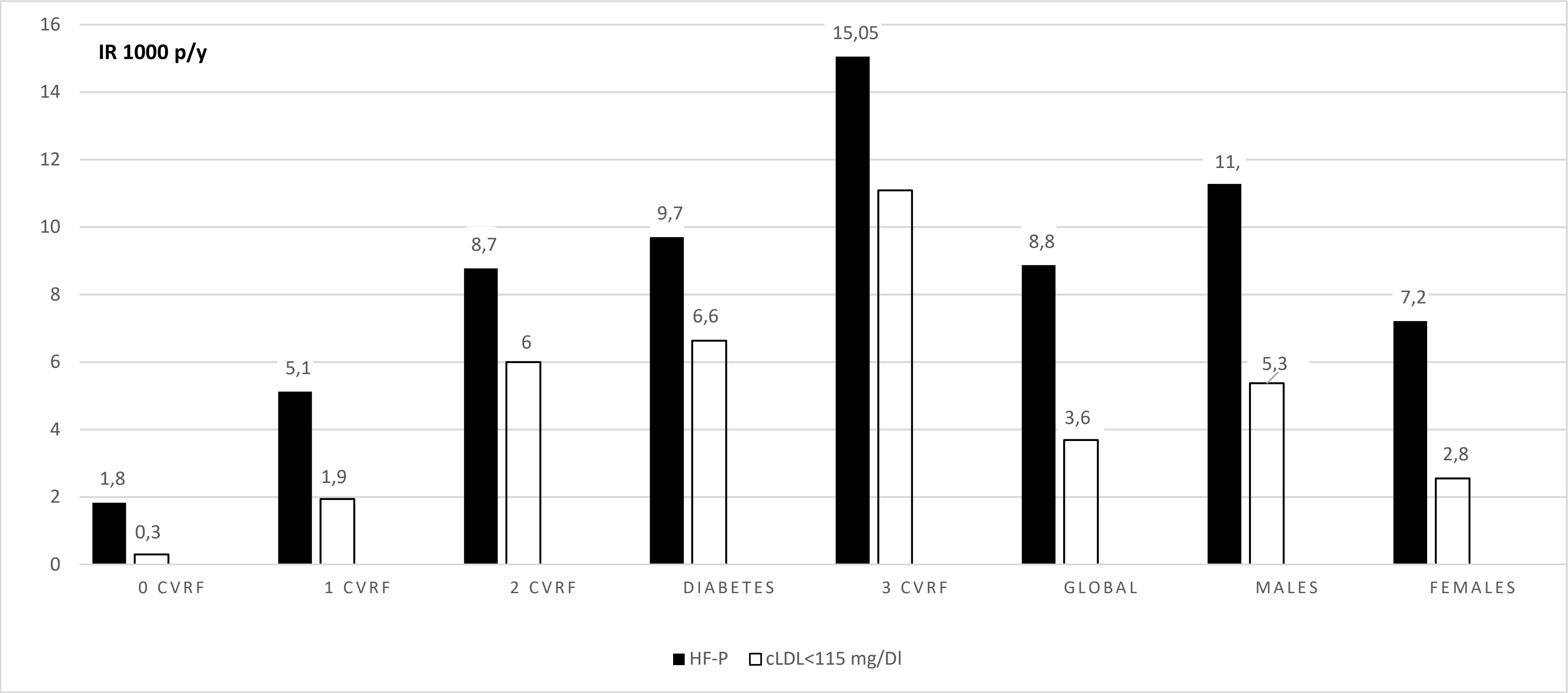

CHD: Coronary Heart Disease; CVRF: Cardiovascular Risk Factor; FH-P: Familial Hypercholesterolemia Phenotype; LDL-C: Low-Density Lipoprotein Cholesterol

Fig S2. Incidence rate (per 1000 person-years) of CHD in secondary prevention in FH-P vs normolipidemic population based on the presence of number of cardiovascular risk factors and clinical associated characteristics

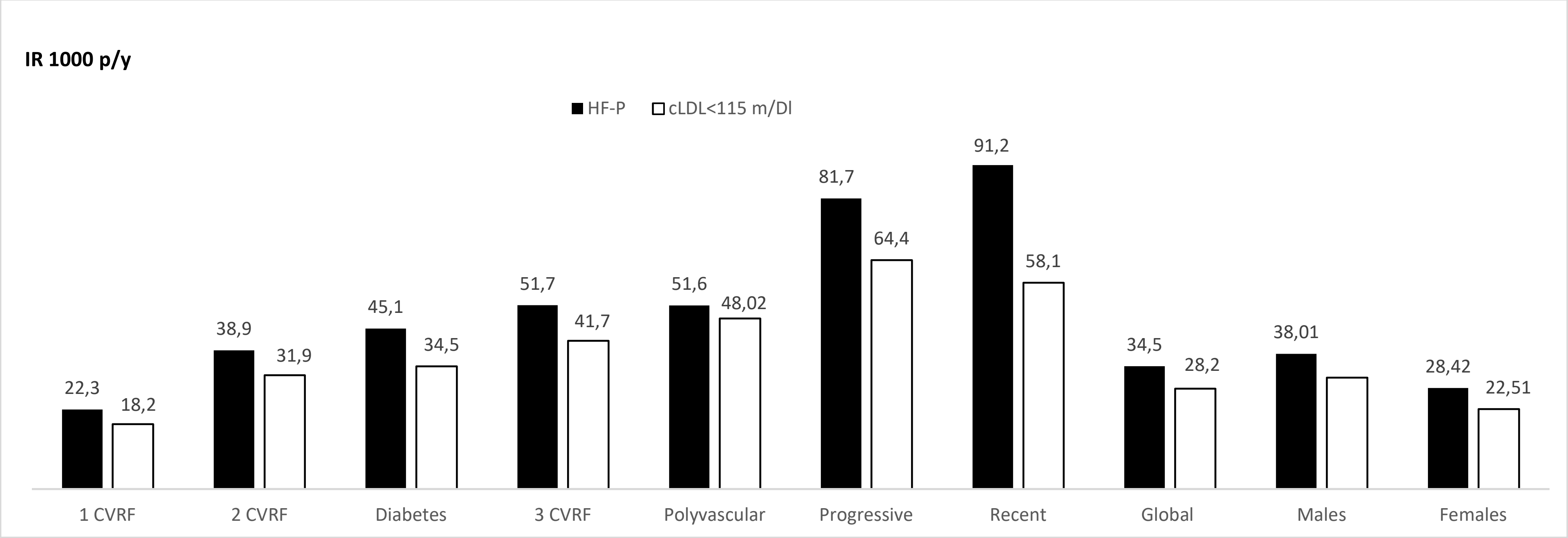

CHD: Coronary Heart Disease; CVRF: Cardiovascular Risk Factor; HF-P: Familial Hypercholesterolemia; CLDL: Cholesterol-LDL

Coronary heart disease

ICD 9

410,
4100, 41000, 41001, 41002,
4101, 41010, 41011, 41012,
4102, 41020, 41021, 41022,
4103, 41030, 41031, 41032,
4104, 41040, 41041, 41042,
4105, 41050, 41051, 41052,
4106, 41060, 41061, 41062,
4107, 41070, 41071, 41072,
4108, 41080, 41081, 41082,
4109, 41090, 41091, 41092,
411, 4110, 4111, 4118, 41181, 41189
413, 4130, 4131, 4132, 4133, 4138, 4139
4140, 41400, 41401, 41402, 41403, 41404, 41405, 41406, 41407

ICD 10

I20, I20.0, I20.1, I20.8, I20.9
I21, I21.0, I21.1, I21.2, I21.3, I21.4, I21.9
I22, I22.0, I22.1, I22.8, I22.9
I23, I23.0, I23.1, I23.2, I23.3, I23.4, I23.5, I23.6, I23.8
I24, I24.0, I24.1, I24.8, I24.9

Ischemic stroke

ICD 9

433,
4330, 43301, 4331, 43311, 4332, 43321, 4333, 43331, 4338, 43381, 4339, 43391
434,
4340, 43401, 4341, 4342, 4349, 43491
438,
4380,
4381, 43810, 43811, 43812, 43813, 43814, 43819,
4382, 43820, 43821, 43822,
4383, 43830, 43831, 43832,
4384, 43840, 43841, 43842,
4385, 43850, 43851, 43852, 43853,
4386,
4387,
4388, 43881, 43882, 43883, 43884, 43885, 43889,
4389

ICD 10

I63, I63.0, I63.2, I63.3, I63.5, I63.6, I63.8, I63.9
I64
I65, I65.0, I65.1, I65.2, I65.3, I65.8, I65.9

Peripheral artery disease

ICD 9

44020, 44021, 44022, 44023, 44024, 44029,
4403, 44030, 44031, 44032,
4404
4439
44422, 44489

ICD 10

I70, I70.2, I70.8, I70.9
I73, I73.8, I73.9
I74.3
